# Who Pays for Low-GI Yogurt in China? Moderating Roles of Health Orientation and Consumer Knowledge

**DOI:** 10.3390/nu18040643

**Published:** 2026-02-16

**Authors:** Yixin Guo, Leyi Wang, Wenxue Tang, Xiaoou Liu

**Affiliations:** School of Agricultural Economics and Rural Development, Renmin University of China, Beijing 100872, China; guoyixin@ruc.edu.cn (Y.G.); wangleyi777@ruc.edu.cn (L.W.); tangwenxue@ruc.edu.cn (W.T.)

**Keywords:** Low-GI claim, front-of-package labeling, willingness to pay, discrete choice experiment

## Abstract

Background: The Glycemic Index (GI) serves as a critical indicator of carbohydrate quality linked to postprandial glycemic response. As “Low-GI” claims proliferate on front-of-pack labels, it remains unclear how consumers value this complex signal. This study quantifies willingness to pay (WTP) for Low-GI labeling and tests a “motivation–capability” mechanism, positing that health orientation motivates label use, while objective Low-GI knowledge facilitates targeted evaluation across nutritional contexts. Methods: A discrete choice experiment was conducted in China using plain yogurt (*N* = 910). Mixed logit models analyzed how the valuation of the Low-GI claim is moderated by carbohydrate context, health orientation, and objective knowledge. Results: Results indicate a significant average premium for Low-GI labeling, with health orientation acting as a consistent motivational amplifier. Objective knowledge functions as a critical moderator interacting with carbohydrate context, driving label valuation only in specific low- or high-carbohydrate profiles while triggering skepticism in regular carbohydrate ones. Conclusions: These findings suggest that the public health effectiveness of emerging physiological claims depends jointly on consumer motivation and label-specific literacy. Consequently, policy interventions should combine label standardization with targeted education, equipping consumers with the capability to decode the claim’s physiological meaning rather than relying on a generalized health halo.

## 1. Introduction

Functional foods that provide physiological benefits beyond basic nutrition represent a market that has expanded significantly alongside advances in nutritional science and the strategic use of health claims for product differentiation [1]. Within this segment, Low-GI products—designed to attenuate postprandial glycemic excursions and support glycemic management—have gained substantial market traction in recent years [2,3]. Given that Low-GI diets are a proven strategy for enhancing carbohydrate quality and mitigating cardiometabolic risks [4,5,6], their application is particularly urgent in China, home to the world’s largest diabetic population [7].

However, while the “Low-GI” label is supported by a mature scientific infrastructure, its meaning and interpretation in real-world purchasing contexts remain contested. As a complex physiological metric, the GI quantifies postprandial glycemic responses through standardized protocols and international GI tables [3,8], yet it is not a single-ingredient attribute because its realized physiological meaning depends critically on the food matrix, processing, and serving context [3]. This contextual dependence also raises labeling concerns: regulatory-facing reviews caution that GI labeling may be misconstrued as a blanket “healthier” signal, potentially diverting attention from the overall nutrient profile of the product [9]. Consistent with this concern, consumers often struggle to interpret GI concepts without guidance, resulting in substantial heterogeneity in understanding and use [10]. Meanwhile, GI-related research continues to evolve, including efforts to link GI metrics with broader carbohydrate quality indicators and dietary assessment frameworks [11].

Against this backdrop of a mature scientific infrastructure yet contested real-world interpretation, empirical evidence quantifying the market value—specifically consumers’ willingness to pay (WTP)—for Low-GI claims remains scarce. Limited evidence, such as for Low-GI rice, suggests that such claims command a premium primarily when glycemic response is highly salient to the consumer [12]. More importantly, systematic review evidence indicates that responses to functional food claims are heterogeneous and patterned by lifestyle, psychological, and socio-demographic correlates [13]. Building on this broad pattern, we extend the literature by proposing a mechanism suited to complex cues: for Low-GI, valuation is likely to follow a motivation–capability–context pathway. While health-oriented consumers (motivation) are more likely to allocate attention to nutrition cues [14,15], as motivation alone is often insufficient for physiologically complex claims. In these cases, objective knowledge (capability) functions as a cognitive “decoder,” enabling consumers to interpret claims—like GI—that cannot be verified by the nutrient panel alone [4,16]. Finally, this valuation is shaped by cue congruence (context)—the degree to which the surrounding nutrient profile supports a coherent interpretation of the claim [17]. To our knowledge, this integrated pathway has not yet been directly quantified for Low-GI labeling in yogurt.

Given the strong context dependence and interpretive challenges of GI claims, yogurt provides an ideal setting to study how Low-GI labeling is valued at the point of purchase. On one hand, yogurt is widely perceived as a “healthy” carrier, which remains physiologically compatible with Low-GI positioning, and increasingly utilizes multifaceted front-of-pack cues to signal nutritional value [18,19]. On the other hand, a substantial body of research has examined consumer demand for functional dairy, spanning general perceptions [20], attribute-specific WTP [21], product design [22], and systematic reviews of preferences [23]. Together, these studies suggest that consumers’ valuations of functional dairy are sensitive to the specific claims and the product’s context. Yet Low-GI yogurt has been largely overlooked as a distinct functional subtype. In existing dairy research, Low-GI is predominantly treated as a physiological property rather than a market signal whose economic value can be monetized [18]. Furthermore, while labeling debates often address compliance and trust [24], they provide limited evidence on the magnitude of a Low-GI premium. In sum, the Low-GI label premium in the yogurt market has yet to be quantified, much less examined through the joint roles of consumer motivation, objective knowledge, and nutritional-context congruence.

To address these gaps, this study employs a discrete choice experiment (DCE) in a yogurt choice setting in China to estimate the willingness to pay (WTP) for Low-GI labeling and to identify systematic preference heterogeneity. DCEs provide a robust framework for decomposing choices into part-worth utilities and testing interaction-based mechanisms under controlled information presentation [25,26,27]. Our approach also aligns with recent consumer research that uses choice experiments to quantify label valuation and preference heterogeneity in food markets [28,29,30]. Empirically, we document a significant average WTP premium for Low-GI labeling and substantial preference heterogeneity. Specifically, health orientation and objective Low-GI knowledge moderate both the overall valuation of the label and its sensitivity to carbohydrate-context differences.

This study contributes to the extant literature in three distinct ways. First, it extends evidence on the WTP for relatively novel and conceptually abstract physiological claims (Low-GI) within a realistic choice setting. In contrast to prior nutrition labeling research that has largely focused on consumer responses to simpler nutrient information and familiar health/credence cues [31,32,33], existing evidence on Low-GI valuation remains relatively limited and concentrated in specific product contexts and stated preference applications [12,34]. Second, it clarifies the role of consumer motivation by quantifying how health orientation shapes functional label valuation, demonstrating that health orientation acts as a consistent amplifier of WTP. Third, it incorporates objective Low-GI knowledge to probe the mechanisms underlying context-dependent valuation, showing that carbohydrate-context variation in the WTP premium is concentrated among knowledgeable consumers—consistent with a decoder-based interpretation of how consumers process complex physiological claims.

## 2. Conceptual Background and Hypotheses Development

### 2.1. Labels as Signals Under Information Asymmetry

A core premise in food labeling research is that when consumers cannot fully verify quality or health outcomes prior to purchase, labels function as signals to mitigate information asymmetry. In economic terms, many health and functional attributes are classified as “credence attributes,” the veracity of which remains difficult to verify even after consumption [35]. In such markets, the interplay between regulatory disclosure and voluntary claims shapes “believable quality cues,” which in turn dictate consumer choice and corporate product strategy [36].

A growing body of valuation evidence further suggests that nutrition and health-related claims can generate measurable willingness to pay (WTP) premia, as consumers leverage these claims as heuristic shortcuts for benefits that are otherwise hard to verify [37,38]. This logic is particularly salient within functional food categories. For instance, empirical studies on functional dairy products document positive WTP for health-enhancing attributes and related claims, while also highlighting substantial preference heterogeneity among consumer segments [38]. Moreover, emerging evidence in Low-GI-related contexts indicates that consumers are willing to pay a premium for GI-oriented health cues when glycemic outcomes are perceived as salient [12]. Collectively, these findings imply that if the Low-GI label is perceived as a credible and informative signal in our yogurt choice setting, it should yield positive marginal utility and a WTP premium relative to unlabeled alternatives. Therefore, we propose the following hypothesis:

**Hypothesis** **1 (H1).** *Consumers derive positive marginal utility from the Low-GI label in our yogurt choice setting, resulting in a premium in WTP relative to products without the label.*

### 2.2. Consumer Heterogeneity: Health Orientation as Motivation

A well-established body of literature posits that consumers do not constitute a homogeneous group; rather, the transition from “encountering a label” to “perceiving value” is fundamentally shaped by motivational disparities. Health orientation (or health consciousness) is widely conceptualized as a stable motivational variable that heightens attentiveness toward health-related cues and increases the likelihood of selecting products featuring health claims [39,40]. In routine purchase environments, this motivational predisposition renders health-oriented consumers more inclined to employ health cues as heuristics when constructing value judgments.

Low-GI front-of-pack claims are typically communicated as a health-oriented cue (i.e., a consumer-facing signal of carbohydrate quality and glycemic management), and thus can function as a health-relevant signal at the point of purchase [41]. Accordingly, consumers with a stronger health orientation should exhibit greater responsiveness to this Low-GI signal. Consequently, even when conditioning on objective Low-GI knowledge, health orientation is expected to amplify the marginal utility derived from the Low-GI label. Based on this reasoning, we propose the following hypothesis:

**Hypothesis** **2 (H2).** *Consumers with higher health orientation place a greater value on the Low-GI label in our yogurt choice setting, independent of their objective Low-GI knowledge. This reflects the motivational aspect of consumer choice, where health-oriented individuals are more prone to use health cues as heuristic signals of “better-for-you” quality.*

### 2.3. Nutritional Context and Congruence

A prevalent argument in food consumption research is that the efficacy of a label is not static but rather contingent upon its congruence with objective nutritional facts and the broader context in which the claim is evaluated. When consumers extrapolate overall healthfulness from a salient claim, this heuristic judgment often manifests as a “health halo” effect. Extensive experimental evidence indicates that health and nutrition claims can significantly enhance consumer perceptions of overall healthfulness—and even modulate risk perception and trust—yet these effects may be discounted or met with skepticism in the presence of informational conflict [42,43]. More granular insights from package information studies suggest that when the nutrition facts panel aligns with the front-of-pack claim (congruence), consumers are more likely to form cohesive positive evaluations. Conversely, in the face of inconsistency, consumers often fail to reconcile the discrepancy or fall prey to heuristic biases, processing the claim in isolation while disregarding the actual nutrition facts [44,45,46].

At the attentional level, visual engagement with nutrition information is further moderated by label presentation and consumer familiarity; thus, in real-world purchase environments, the synergy of “FOP signals + key nutrition facts” serves as a more powerful determinant of choice than either source alone [17,47,48]. Collectively, these findings imply that identifying the true market value of a complex claim like Low-GI requires empirical testing within a specific nutritional context rather than estimation in isolation. To capture these dynamics, we explicitly model the interaction between the Low-GI label and carbohydrate content. This approach enables us to operationalize the theoretical constructs of “congruence” and “halo effects” into an estimable utility structure, thereby testing whether consumers calibrate their valuation based on nutritional consistency.

If the diagnosticity of a health claim is conditional upon its alignment with surrounding nutrition cues, the Low-GI premium should fluctuate across carbohydrate contexts rather than remain invariant. Accordingly, the marginal utility of the Low-GI label is expected to be amplified when the product’s carbohydrate profile provides a more coherent rationale for the claim. Therefore, we propose the following hypothesis:

**Hypothesis** **3 (H3).** *The valuation of the Low-GI label in our yogurt choice setting is context-dependent; its utility is amplified when the product’s carbohydrate context aligns with the label to present a more coherent health scenario (e.g., low-carbohydrate contexts).*

### 2.4. Nutrition Knowledge as a “Decoder”

Nutrition knowledge and literacy play a critical role in determining whether consumers can effectively synthesize on-pack information, thereby moderating the marginal effect of the claim [33]. From the perspective of labeling economics, the efficacy of a signal is fundamentally dictated by its verifiability, the cognitive cost of comprehension, and its synergy with supplementary quality cues [49]. This is particularly salient for complex functional signals like Low-GI, which lack a direct correspondence to intuitive nutritional dimensions (e.g., “low fat” or “low sugar”) and instead require specific expertise to map “GI” onto tangible health outcomes. Consequently, the signaling strength of Low-GI is likely contingent upon consumer cognitive processing depth and trust in the information source—a notion supported by cross-national evidence revealing substantial disparities in the perceived credibility and acceptance of health claims [50]. Classic frameworks of consumer expertise further suggest that knowledge recalibrates the weighting of extrinsic cues, enabling high-knowledge individuals to execute consistency checks and reduce over-reliance on isolated signals [51]. Building on these perspectives, our study operationalizes a “decoder” mechanism by examining whether objective Low-GI knowledge empowers consumers to discern the functional implications of the label across varying nutritional contexts.

If context-dependent valuation requires the integration of claims with factual data, this process should be more robust among knowledgeable consumers. Thus, objective Low-GI knowledge should facilitate differentiated valuation—strengthening WTP where the label is diagnostic and discounting it where the context remains ambiguous. Therefore, we propose the following hypothesis:

**Hypothesis** **4 (H4).** *Objective Low-GI knowledge acts as a cognitive decoder that enables consumers to distinguish the functional mechanism of the label across contexts. Knowledgeable consumers will not value the label uniformly; instead, they will exhibit higher WTP in contexts where the label offers a clearer functional rationale—either as perceived two-cue reinforcement (low-carbohydrate contexts) or as a cue for a more gradual postprandial glycemic response relative to higher-GI alternatives (high-carbohydrate contexts)—while discounting the label in ambiguous regular-carbohydrate contexts where the functional positioning is less diagnostic.*

## 3. Materials and Methods

### 3.1. Survey Administration and Participants

The study was carried out online in late 2025 (November–December) via the Wenjuanxing platform (Changsha Ranxing Information Technology Co., Ltd., Changsha, China). Participation was voluntary and anonymous. Before proceeding, respondents were informed that the survey was for academic research purposes and provided informed consent. Participants were recruited via posts containing a survey link shared in WeChat groups (WeChat, Tencent, Shenzhen, China), using a non-probability convenience sampling approach [52,53]. Eligible participants were screened at the beginning of the questionnaire and were required to be 18 years or older and to be the primary or joint household food purchase decision-maker. Respondents who completed the survey received a small cash incentive.

To ensure data quality, the Wenjuanxing platform was configured to restrict multiple submissions from the same device. In addition, an instructional attention-check item was embedded in the questionnaire. Responses that failed this attention check (which instructed respondents to select “Agree”) were excluded from the analysis. We further removed responses with completion time shorter than 4 min to reduce rapid satisficing. The final analytic sample consisted of 910 valid respondents, contributing 10,920 choice tasks (910 × 12). The average completion time was approximately 6.7 min, suggesting limited rapid satisficing. These procedures are consistent with common practices in web-based surveys using social network distribution in China, where survey links are disseminated via WeChat and unreliable cases are explicitly removed [53,54], and with web-based stated preference studies that document recruitment via online networks and quality screening [55].

The online questionnaire consisted of five parts. First, respondents completed informed consent and eligibility screening (age ≥ 18; being the primary or joint household food purchase decision-maker). Second, we elicited category-related background information on original-flavored yogurt purchase habits and reference information, including purchase frequency, nutrition label viewing, typical price range (per 100 g), usual package size, purchase channels, and consumption occasions. Third, respondents completed the discrete choice experiment (DCE), which is described in detail in the following section. Fourth, immediately after the DCE, respondents answered an attribute non-attendance (ANA) check, indicating whether any attribute (Low-GI label, carbohydrate content, fat type, organic label, or price) was ignored when making choices. Fifth, respondents completed measures on Low-GI-related knowledge, perceptions of label meaning and “health halo” beliefs, label trust, health orientation, taste/texture importance, and socio-demographic characteristics; an instructional attention check (“please select ‘Agree’”) was embedded to ensure response quality.

### 3.2. Discrete Choice Experiment (DCE): Attributes and Design

The study employed a generic discrete choice experiment to elicit consumer preferences for original-flavored yogurt. Each choice task presented three product alternatives (A/B/C) and an opt-out option (“I would not buy any of these/I would buy other brands”), which avoids forced choices and better reflects real purchase settings. A short cheap-talk script was presented prior to the DCE to encourage respondents to make choices as if they were shopping in a real-world setting, considering their actual budget and habits.

Each yogurt alternative was described by five attributes. Specifically, these attributes included price, carbohydrate content, fat content, the Low-GI label, and the organic label. Although not central to our research question, fat content and the organic label were included as market-relevant controls to capture salient health/quality cues and reduce confounding when estimating preferences for the Low-GI label [56,57]. Except for price, all attributes were categorical and subsequently represented by dummy variables in the econometric models. To reduce information asymmetry and help respondents better anchor their choices, the levels for nutritional attributes (carbohydrate and fat content) were explicitly presented with auxiliary numerical values in addition to their categorical labels. Carbohydrate content, rather than sugar, was selected because Chinese nutrition fact panels commonly report carbohydrates as a standard item, whereas sugar information is not consistently displayed; moreover, in yogurt products, carbohydrate levels are closely related to perceived sweetness and energy density, providing a familiar and comparable nutrition reference for consumers.

**Figure 1:**
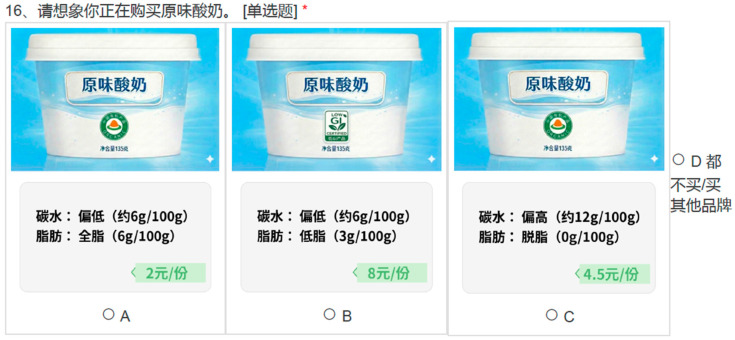
An example of a decision situation. The original questionnaire was designed in Chinese; an English version of this example is provided in Appendix A Figure A1 for reference. * indicates a mandatory question.

Prior to the DCE, respondents were shown the corresponding package labels and informed that the products carried “Low-GI” and “organic” labels, but no further explanation of the Low-GI concept was provided. This was intentional to avoid priming or experimenter demand effects and to preserve the naturally existing variation in consumers’ knowledge. After completing the DCE, respondents took an objective knowledge quiz about Low-GI, and the measured level of understanding was used as a key source of heterogeneity in subsequent analyses. The specific attributes and levels are presented in Table 1. A sample choice card illustrating the presentation of these categorical labels and auxiliary values is shown in Figure 1.

To determine the price levels used in the discrete choice experiment, we conducted a market scan of posted prices for high-selling plain/original yogurt products on major Chinese e-commerce platforms (JD.com, Beijing, China; Taobao, Hangzhou, China) in late November and early December 2025. We identified best-selling SKUs based on category ranking pages and sales-sorted search results, and recorded the displayed selling price and net weight for approximately 50 products across single cups and multipacks. To align the market information with the choice-card presentation, observed prices were converted into a per-135 g equivalent. We used the on-page selling prices observed at the time of scanning and did not rely on extreme, short-lived promotion-only outliers. The resulting per-135 g equivalent prices indicated a mainstream band around approximately CNY 3.8–4.9, occasional promotional lows around CNY 2, and premium products (e.g., Greek-style) reaching around CNY 8 per 135 g. Based on this observed market range, we set three price levels (CNY 2, 4.5, and 8 per 135 g) to represent a discounted/economy option, a mainstream market-centered option, and an upper-end premium option, respectively.

A pilot study (*n* ≈ 80) was conducted to test comprehension and to obtain preliminary parameter estimates. Based on the pilot results, a Bayesian D-efficient design was generated using NGENE v1.1.1 (ChoiceMetrics, Sydney, Australia), with normally distributed priors specified using pilot-based means and variances. During the design generation process, specific constraints were implemented to prohibit dominant alternatives. The final experimental design comprised 24 choice sets, which were divided into two blocks of 12 tasks each to reduce respondent burden. Each participant was randomly assigned to one block and completed 12 choice tasks.

### 3.3. Variable Construction

To examine preference heterogeneity related to health motivation and objective understanding of the Low-GI concept, two measures were constructed from the questionnaire. Health orientation was assessed using three 7-point Likert items: “I often reflect on my health status”, “I am very sensitive to changes in my health”, and “I actively pay attention to the impact of my daily diet on my health”. Responses were averaged to form a composite index and mean-centered prior to inclusion in interaction terms with the Low-GI label. The scale showed good internal consistency (Cronbach’s α = 0.856). Construct validity was supported (KMO = 0.733; Bartlett’s test *p* < 0.001), and an exploratory one-factor solution yielded strong loadings (0.872–0.891), with AVE = 0.778 and CR = 0.913.

Objective knowledge was conceptualized as factual understanding of the core physiological meaning of the Low-GI concept, i.e., whether respondents can correctly distinguish glycemic response rate/trajectory from carbohydrate quantity—a distinction central to interpreting Low-GI claims. This approach follows the consumer knowledge literature that distinguishes objective knowledge (accuracy-based) from familiarity or perceived knowledge [58,59], and the nutrition knowledge measurement tradition that commonly operationalizes objective knowledge using brief factual quiz items assessing concept recognition [16,60]. Importantly, to avoid priming and experimenter demand effects, no additional explanation of Low-GI was provided before choice tasks; instead, the knowledge check was administered after the DCE so that naturally occurring variation in knowledge could serve as an exogenous source of heterogeneity.

In the questionnaire, objective Low-GI knowledge was measured using a multiple-choice definition item asking respondents to select the most accurate statement about Low-GI. The scientifically correct option defined Low-GI as implying a more gradual postprandial blood glucose rise given the same carbohydrate intake. Alternative options captured common misconceptions—interpreting Low-GI as (i) having lower carbohydrate/sugar content, (ii) involving less carbohydrate/sugar absorption by the body, or (iii) “do not know/unsure.” Respondents choosing the correct definition were coded as ObjKnow = 1, and all other responses were coded as ObjKnow = 0. This operationalization therefore captures objective concept recognition rather than breadth or depth of nutritional knowledge. We adopted a single-item objective check because the construct is narrow and unidimensional in the present context, and because keeping the quiz brief reduces cognitive burden and satisficing in survey-based stated preference tasks [61,62].

### 3.4. Econometric Specification and Model Strategy

#### 3.4.1. Random Utility Framework

Consumer choices were modeled within the Random Utility Theory (RUT) framework. For a respondent n in choice situation t, the utility derived from selecting alternative j among J options (comprising three yogurt profiles and an opt-out option) is expressed as follows:

$$U_{njt}=V_{njt}+\varepsilon_{njt}$$

where $V_{njt}$ represents the deterministic component of utility and $\varepsilon_{njt}$ is the stochastic error term, assumed to be independently and identically distributed (i.i.d.) following a Gumbel (Type I extreme value) distribution. The systematic utility of the opt-out option was normalized to zero. A single alternative-specific constant ($ASC_{buy}$) was specified for the three purchase alternatives, with opt-out as the reference, to capture the baseline propensity to purchase a yogurt product.

#### 3.4.2. Mixed Logit Estimation

To account for unobserved preference heterogeneity, mixed logit (MIXL) models were estimated using simulated maximum likelihood. The vector of taste parameters $\beta_{n}$ is assumed to vary across individuals according to a multivariate normal distribution:

$$\beta_{n}=\beta +\eta_{n},\eta_{n}∼N\left(0,\Sigma \right)$$

where β is the vector of mean population preferences and Σ is the covariance matrix. To capture the correlations among random parameters, a Cholesky decomposition was applied to the covariance matrix. In our model specification, six attribute parameters—the Low-GI label, organic certification, two dummy variables for carbohydrate levels (low and high), and two dummy variables for fat content (skim and whole)—were specified as random parameters following a normal distribution. Conversely, the price coefficient, the alternative-specific constant ($ASC_{buy}$), and all interaction terms (including two-way and three-way interactions) were treated as fixed parameters. The price coefficient was kept fixed to ensure stability in the estimation of willingness to pay (WTP) and to avoid the identification issues associated with a random price distribution. Interaction terms were fixed to facilitate a more straightforward interpretation of how specific consumer characteristics (health orientation and knowledge) moderate the utility of the Low-GI label. The models were estimated using 500 Halton draws in Stata 16.0 (StataCorp LLC, College Station, TX, USA).

#### 3.4.3. Model Sequence

Three sequential models were estimated to test the research hypotheses (H1–H4). For categorical attributes; “regular carbohydrate” and “low-fat” were set as the reference categories.

Model 1 (Baseline Main Effects): This model evaluates the general impact of the Low-GI label and other product attributes. The systematic utility is defined as follows:

$$V_{njt}^{\left(1\right)}=\mathrm{AS}C_{\mathrm{buy}}\cdot I\left(j\neq 0\right)+\beta_{p}\cdot {price}_{njt}+\beta_{GI}\cdot {lowGI}_{njt}+\beta_{org}\cdot {organic}_{njt}+\beta_{cl}\cdot {carb\_low}_{njt}+\beta_{ch}\cdot {carb\_high}_{njt}+\beta_{fs}\cdot {fat\_skim}_{njt}+\beta_{fw}\cdot {fat\_whole}_{njt}$$

H1 is supported if $\beta_{GI}>0$ and is statistically significant. The mean WTP for the Low-GI label is computed as $-\beta_{GI}/\beta_{p}$.

Model 2 (Two-Way Interaction): To investigate how Low-GI valuation is moderated by health orientation, carbohydrate context, and objective knowledge, interaction terms were introduced:

$$V_{njt}^{\left(2\right)}=V_{njt}^{\left(1\right)}+\gamma_{L}\cdot {lowGI}_{njt}\cdot {carb\_low}_{njt}+\gamma_{H}\cdot {lowGI}_{njt}\cdot {carb\_high}_{njt}+\delta \cdot {lowGI}_{njt}\cdot {health}_{n}+\kappa \cdot {lowGI}_{njt}\cdot {objknow}_{n}$$

H2 is assessed via the interaction between the Low-GI label and health orientation scores, while H3 is tested through the interactions with carbohydrate levels.

Model 3 (Three-Way Interaction): To explore the “decoder” role of knowledge (H4), we included three-way interaction terms:

$$V_{njt}^{\left(3\right)}=V_{njt}^{\left(2\right)}+\theta_{L}\cdot {lowGI}_{njt}\cdot {carb\_low}_{njt}\cdot {objknow}_{n}+\theta_{H}\cdot {lowGI}_{njt}\cdot {carb\_high}_{njt}\cdot {objknow}_{n}$$

This model tests whether objective knowledge conditions the cue congruence effect of Low-GI and carbohydrate levels. By combining these coefficients, the marginal utility and WTP for Low-GI products can be derived across different carbohydrate contexts and knowledge groups.

## 4. Results

### 4.1. Sample Characteristics and Descriptive Statistics

The socio-demographic profile of the valid respondents (N=910) is summarized in Table 2. The sample exhibited a balanced gender distribution, with 50.11% males and 49.89% females. In terms of age, the largest groups were aged 18–25 (29.34%) and 26–35 (21.65%), representing a relatively young and active consumer segment. Regarding education, the majority of participants had completed vocational or higher education, with 19.78% holding a bachelor’s degree. The geographic distribution was diverse, with a broad representation across different regions and city tiers, ranging from Tier-1 cities (15.38%) to rural areas (17.80%), ensuring a wide coverage of the Chinese consumer market.

Regarding economic status, 53.63% of households reported a monthly post-tax income of less than 5000 CNY, while 25.82% earned between 5000 and 9999 CNY. In terms of consumption habits, over 60% of respondents purchased original-flavored yogurt at least twice a month, with 51.76% buying it 2–3 times monthly, indicating high familiarity with the product category. Notably, in the discrete choice experiment, the opt-out option (“would not buy”) was selected in only 7.44% of the total choice tasks. This low opt-out rate suggests that the experimental design successfully presented realistic and attractive alternatives, and that respondents were highly engaged in the decision-making process.

In addition, respondents’ health orientation and objective Low-GI knowledge were summarized as key individual characteristics. The mean health orientation index (average of three items on a 1–7 scale) was 4.201 (SD = 1.510), suggesting moderate health-related attentiveness in the sample. Regarding objective Low-GI knowledge, fewer than half of the respondents correctly identified Low-GI as indicating a more gradual postprandial blood glucose rise under the same carbohydrate intake (46.48%). Misconceptions were common: 28.13% interpreted Low-GI as implying lower carbohydrate/sugar content, and 20.00% believed it means that less carbohydrate/sugar is absorbed by the body, while 5.38% reported not knowing the definition. This distribution highlights substantial knowledge gaps and supports our focus on knowledge as a key factor shaping how consumers interpret Low-GI information in different carbohydrate contexts.

### 4.2. General Consumer Preferences: Baseline Results (Model 1)

Model 1 in Table 3 presents the baseline mixed logit (MIXL) estimation results, which evaluate consumer preferences for the primary attributes of original-flavored yogurt. Consistent with economic theory, the coefficient for price is negative and statistically significant (p<0.01), indicating that higher price levels decrease the probability of selection. The alternative-specific constant ($ASC_{buy}$) is positive and highly significant, reflecting a robust baseline propensity among respondents to select one of the yogurt alternatives rather than the opt-out option, thereby suggesting a high level of engagement with the product category.

Regarding the labeled attributes, the Low-GI label yields a positive and highly significant utility premium (β=0.331,  p<0.001). This result demonstrates that consumers derive substantial marginal utility from the presence of a Low-GI claim relative to an otherwise identical product, providing strong empirical support for H1. Similarly, the organic label exerts a positive and significant effect on choice, aligning with the previous literature that identifies organic certification as a key credence attribute associated with perceived healthfulness and superior quality.

In contrast, the preference for physical attributes reveals a different pattern. Relative to the reference level (low-fat), both skim and whole-fat alternatives are associated with significantly lower utility. This implies that respondents in this study systematically favor the baseline “low-fat” profile over the extremes of fat content in the context of original-flavored yogurt. Regarding carbohydrate content, respondents exhibited a significant preference for the low-carbohydrate profile (β=0.175,  p<0.01) relative to the regular baseline, whereas the high-carbohydrate alternative did not yield a statistically significant difference in utility.

Finally, the estimated standard deviations of the random coefficients are statistically significant for all key attributes. This confirms the presence of substantial unobserved preference heterogeneity across the sample, validating the use of the mixed logit specification to capture individual-level variations in the valuation of yogurt attributes and functional labels.

### 4.3. Moderating Effects of Context and Individual Characteristics (Models 2 and 3)

Column 2 in Table 3 presents the estimation results for Model 2, which extends the baseline specification by introducing several two-way interaction terms to examine whether the utility derived from the Low-GI label is conditional upon the product’s carbohydrate context and the consumers’ individual characteristics. The empirical results reveal distinct and statistically significant moderation patterns that refine the understanding of Low-GI label valuation. The model captures the role of individual heterogeneity through health orientation moderation (H2). The interaction between the Low-GI label and health orientation is positive and statistically significant ($\beta_{LowGI\times health}=0.075,\;p=0.001$). Given that the health orientation variable is mean-centered, this estimated coefficient indicates that respondents with a health orientation above the sample average place a higher valuation on the Low-GI label, a finding that is consistent with the predictions of H2. This suggests that consumers who are more intrinsically motivated by health-related goals are more sensitive to functional claims that facilitate the selection of healthier dietary options.

Furthermore, regarding context moderation (H3), the interaction between the Low-GI label and the low-carbohydrate yogurt profile is positive and highly significant ($\beta_{LowGI\times carb\_low}=0.271,\;p<0.001$), indicating that the marginal utility of a Low-GI claim is substantially amplified when the product is presented within a low-carbohydrate nutritional context. In contrast, the interaction between the Low-GI label and the high-carbohydrate profile does not reach statistical significance, suggesting that the incremental value of the Low-GI label does not significantly differ in a high-carbohydrate context relative to the reference carbohydrate level. Taken together, these findings provide empirical support for H3, suggesting that the effectiveness of a functional label is context-dependent and appears stronger when the overall nutritional profile is conceptually congruent with the health cues signaled by the label.

The interaction between the Low-GI label and objective knowledge (objknow) is not statistically significant in Model 2, implying that knowledge does not act as a simple additive moderator of Low-GI valuation in a generalized context. This lack of a direct interaction effect motivates the subsequent analysis of higher-order interactions in Model 3. In this final specification, objective knowledge is examined as a potential “decoder” that shapes how consumers jointly interpret the Low-GI claim and the specific carbohydrate context provided. This approach allows for a more nuanced investigation of whether nutritional knowledge is required to reconcile functional information with the physical attributes of the product, particularly when such information may appear complex or contradictory to uninformed consumers.

It is also important to highlight the robustness of the model specification. As shown in Table 3, the coefficients for the non-interacted control attributes—specifically price, organic certification, and fat content—remained remarkably stable in magnitude and significance compared to the baseline model. While the main effect of the low-carbohydrate attribute decreased in magnitude in Model 2, this is an expected structural shift: the utility previously attributed to the low-carbohydrate attribute alone is now captured by the significant interaction term ($\beta_{LowGI\times carb\_low}$). This pattern clarifies the interpretation of the coefficients: once the interaction is included, the main effect is identified at the baseline (Low-GI = 0), while the significant interaction term captures the incremental utility of low carbohydrate specifically when the label is present, consistent with H3.

### 4.4. The “Decoder” Role of Knowledge

Column 3 in Table 3 presents the estimation results of Model 3, which extends the analysis by incorporating three-way interaction terms between objective knowledge, the Low-GI label, and carbohydrate levels. This advanced specification is explicitly designed to test H4 by examining whether nutritional knowledge functions as a cognitive “decoder”, enabling consumers to discriminate the value of the Low-GI label based on the specific carbohydrate context. The empirical results provide robust evidence that knowledgeable consumers do not value the label uniformly; rather, they adjust their preference according to the distinct nutritional logic of each product profile, distinguishing between the physiological velocity of the glucose response (GI) and the total quantity of carbohydrate intake.

The most pronounced positive effect is observed in the low-carbohydrate context ($\beta_{obj\times LowGI\times carb\_low}=0.617,\;p<0.001$). For knowledgeable consumers, this combination is consistent with a cue-reinforcing interpretation of quantity and quality cues. Specifically, the low-carbohydrate attribute provides a favorable quantity signal (lower carbohydrate intake), while the Low-GI label provides a favorable quality signal (slower glycemic response rate). Together, the pattern of estimates suggests that the marginal value of the Low-GI label is highest when the surrounding nutritional information supports a coherent physiological narrative. Notably, comparing Model 2 and Model 3 reveals a critical insight regarding the base interaction effect. Once objective knowledge is explicitly modeled in the three-way interaction, the two-way interaction term between the Low-GI label and the low-carbohydrate context (Low-GI × Carb: low) becomes statistically insignificant (β=−0.014, p>0.10). In Model 3, this term specifically captures the incremental effect among less knowledgeable consumers (ObjKnow = 0). Its insignificance suggests that, for consumers who do not correctly understand the Low-GI concept, the Low-GI claim does not generate additional utility in the low-carb setting beyond the main effects. This pattern is consistent with the possibility that low-knowledge consumers do not systematically integrate the Low-GI claim with carbohydrate quantity cues; instead, they may treat the two pieces of information as weakly diagnostic or only loosely related. This pattern clarifies that the cue congruence effect originally detected in Model 2 is, in fact, driven almost entirely by the knowledgeable segment. By isolating this effect, Model 3 further supports the proposed “decoder” role of knowledge, showing that the cue-reinforcement mechanism in perceived healthfulness operates only when consumers have sufficient cognitive capabilities to interpret the signal.

Crucially, a positive and significant effect also emerges in the high-carbohydrate context ($\beta_{obj\times LowGI\times carb\_high}=0.307,\;p<0.001$), although the magnitude is smaller than in the low-carbohydrate scenario. In the high-carbohydrate context, the positive three-way interaction is consistent with a risk-mitigation interpretation: knowledgeable consumers may interpret the Low-GI label as a cue of slower carbohydrate absorption, leading them to expect a more gradual postprandial glycemic response relative to higher-GI alternatives, even in a high-carbohydrate option [5,6]. More broadly, the estimates suggest that objective knowledge is associated with greater reliance on a mechanism-based evaluation (GI as a “rate” cue) rather than a purely heuristic “health halo.”

In contrast, relative to these specialized profiles, the valuation of the Low-GI label in the regular-carbohydrate context (the reference category) is significantly lower. Crucially, the negative interaction term observed here does not imply that knowledgeable consumers view the label negatively; rather, it indicates that their net marginal utility in the reference context (calculated as $\beta_{LowGI}+\beta_{LowGI\times ObjKnow}$) converges toward zero, rendering the WTP statistically insignificant. For knowledgeable consumers, a “regular carb + Low-GI” product lacks clear functional positioning, being neither a strict diet option nor a specialized energy source. Consequently, without the clear cue congruence in perceived healthfulness (in low-carbohydrate contexts) or risk-mitigation value (in high-carbohydrate contexts) found at the extremes, the marginal utility of the label diminishes. Collectively, these findings support the view that objective knowledge acts as a sophisticated filter that amplifies the label’s value when it offers a clearer perceived functional rationale, thereby providing strong empirical support for H4.

Finally, the stability of the estimates across Model 2 and Model 3 warrants mention. Despite the increased complexity introduced by the three-way interaction terms, the coefficients for the control variables (price, organic, and fat) and the carbohydrate main effects remained virtually unchanged compared to Model 2. Specifically, the main effect for the low-carbohydrate attribute remained statistically insignificant (β=0.030, p>0.1), mirroring the result in Model 2. This consistency confirms that for the baseline group (uninformed consumers in the absence of a Low-GI label), physical carbohydrate reduction alone continues to generate limited utility.

### 4.5. Willingness to Pay (WTP) Estimates and Economic Implications

To translate utility estimates into economically interpretable metrics, willingness to pay (WTP) for the Low-GI label was computed as $\mathrm{WTP}=-\Delta U/\beta_{price}$ using the delta method. We calculate WTP for the Low-GI label based on the estimated parameters from Model 3 (Table 3), because it provides the richest representation of preference heterogeneity by incorporating both contextual interactions and individual-level moderators, and it also delivers the best overall fit among the competing specifications, as indicated by the lowest AIC and BIC. Table 4 reports WTP (CNY) across three carbohydrate contexts (low/regular/high), stratified by objective knowledge (Obj = 0 vs. 1) and health orientation (evaluated at 0 and ±1 SD of the mean-centered health index). The results show that the economic value of the Low-GI label is strongly context-dependent and differs systematically across consumer segments.

Health orientation acts as a general upward shift in valuation. Across knowledge groups and carbohydrate contexts, higher health orientation is associated with higher WTP for the Low-GI label. For example, among consumers without objective knowledge in the regular-carbohydrate context, WTP increases monotonically from 2.14 CNY (−1 SD) to 4.55 CNY (+1 SD). This pattern indicates that stronger health motivation translates into a greater willingness to pay for a health-related label.

Objective knowledge operates as a contextual “decoder”, amplifying valuation when carbohydrate information provides a congruent (or salient) context. In the low-carbohydrate context, knowledgeable consumers exhibit a substantially larger premium for the Low-GI label, amounting to nearly 2.3 times the value placed by uninformed consumers (7.42 CNY vs. 3.20 CNY). In the high-carbohydrate context, knowledgeable consumers also maintain a higher WTP (3.58 CNY) than uninformed consumers (2.67 CNY). These differences suggest that objective knowledge helps consumers integrate the Low-GI claim with the product’s carbohydrate profile, thereby strengthening the perceived relevance of the label in context.

In contrast, in the regular-carbohydrate reference context, knowledgeable consumers show a small and statistically insignificant WTP (0.97 CNY), while uninformed consumers display a moderate premium (3.35 CNY). This pattern may reflect differences in information processing: consumers with limited objective knowledge may rely on a general “health halo” associated with the label, whereas knowledgeable consumers may process the label in a more systematic way when the surrounding nutritional context does not clearly signal a low- or high-carbohydrate positioning, making the incremental information content of the label less diagnostic.

Finally, the magnitude of the Low-GI premium is economically meaningful when benchmarked against other attributes. Under the low-carbohydrate context among knowledgeable consumers with higher health orientation (+1 SD), WTP reaches 8.62 CNY, exceeding the premium associated with the organic label (1.60 CNY). This comparison highlights that Low-GI can function as a high-intensity functional signal, but the premium is most likely to be realized when the label is paired with a nutritionally congruent product profile and targeted toward consumers with sufficient nutritional literacy to interpret its physiological implications.

**Figure 2:**
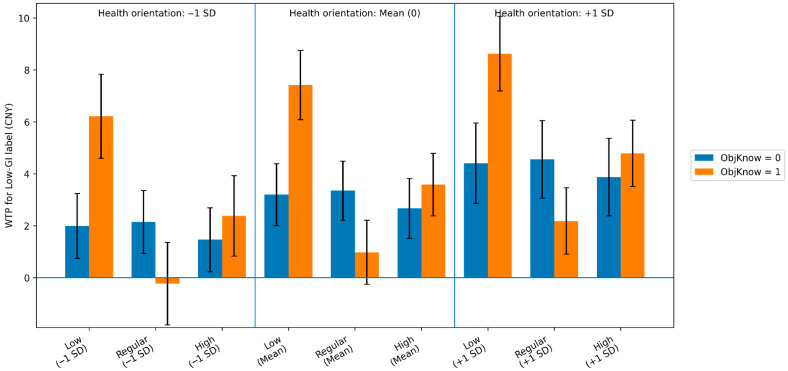
Consumers’ willingness to pay for Low-GI under various health orientations, respective objective knowledge and carbohydrate content. The baseline is “regular carbohydrate”, objective knowledge = 0, and mean health orientation (centered at 0).

Figure 2 visualizes the WTP estimates from Table 4, highlighting three systematic regularities. First, health orientation acts as a consistent amplifier, increasing WTP across all contexts. Second, objective knowledge generates a strong cue congruence premium: in the low-carbohydrate context, knowledgeable consumers exhibit the highest WTP, which widens further with health motivation. Third, a “decoder” mechanism is evident in the distinction between contexts: unlike uninformed consumers who value the label globally, knowledgeable consumers discount the label in the regular-carbohydrate context while maintaining a positive “risk-mitigation” valuation in the high-carbohydrate setting. This confirms that the label’s economic value is structurally determined by the interplay of nutritional context and consumer capability.

## 5. Discussion

### 5.1. General Preference for Low-GI Labeling (Baseline Valuation)

Across the full sample, the Low-GI claim generated a statistically significant utility premium, implying that consumers are willing to pay extra for this FOP signal. This baseline result is consistent with labeling research emphasizing how front-of-pack information can reduce search costs and shape product evaluation when quality/health attributes are not directly observable [63,64]. In the yogurt market specifically, choice and conjoint evidence shows that consumers often attach a positive value to nutrition- and health-related claims and certification-type cues, although valuations vary across segments [65,66,67,68].

Building on this broader functional-dairy evidence, our result also extends the emerging Low-GI valuation literature into a yogurt setting. Prior work that monetizes the willingness to pay for Low-GI (or GI-oriented) cues has largely focused on non-dairy categories—especially rice and other staple-food contexts [12,34,69]. By directly estimating a WTP premium for a Low-GI claim in yogurt, we fill this category gap and provide baseline support for H1: consumers derive positive marginal utility from the Low-GI label relative to otherwise comparable unlabeled products.

### 5.2. The Role of Health Motivation

We find clear evidence that health orientation amplifies consumers’ valuation of the Low-GI claim. This pattern aligns with prior evidence that health motivation and nutrition orientation increase attention to, and use of, on-pack nutrition information [14,15]. It is also consistent with valuation studies showing that more health-conscious consumers tend to place higher premiums on food labels and health-related product attributes, because such cues help them pursue health goals under time and information constraints [70,71].

Importantly, yogurt-focused stated preference studies already document systematic preference heterogeneity in responses to nutrition/health claims and related attributes [65,66,67]. However, this literature has not explicitly linked heterogeneity to a direct, theory-consistent motivational construct such as health orientation in the specific case of a physiologically complex cue like Low-GI. In related non-dairy contexts, GI-oriented WTP studies also report preference heterogeneity, but do not consistently attribute it to health orientation [12,34,69,72]. By explicitly modeling health orientation as a moderator of the Low-GI premium in a yogurt setting, our results complement this literature by identifying a motivation-based channel through which the same GI cue can elicit systematically different WTP responses.

From the perspective of food-labeling interventions, our results imply that the same Low-GI claim can generate markedly different behavioral responses depending on consumers’ motivational state. For policy and industry strategy, increasing label availability alone may not shift choices uniformly; the strongest effects are expected among consumers who already prioritize health goals [15]. Overall, these findings provide direct empirical support for H2, showing that health orientation systematically increases the marginal utility—and thus the WTP premium—associated with the Low-GI label.

### 5.3. The Role of Nutritional Context and Knowledge

A central contribution of this study is showing that the Low-GI claim is not evaluated in isolation. Its value depends on the broader nutritional context—here, carbohydrate content—because consumers interpret multiple on-pack cues jointly. This is consistent with evidence that consumers’ responses to health claims depend strongly on what other nutrition information appears on the package [17] and on how claims interact with nutrition facts and consumers’ motivation to process information [73]. In dairy settings, conjoint evidence on yogurts explicitly incorporates the joint role of health claims and nutritional composition, showing that the valuation of claims differs when the underlying nutrient profile is more versus less favorable [74]. Related experimental evidence using yogurt among other products further suggests that health claims and front-of-pack labels can trigger heuristic inferences (e.g., positivity bias/health halo), and that these effects vary with the product’s objective healthfulness—highlighting why nutrient context matters for interpreting claims [75]. Complementing this demand-side evidence, nutrient profiling evaluations of dairy products indicate that the appropriateness of on-pack claims is inherently tied to overall nutritional composition, reinforcing the centrality of claim–nutrient congruence in dairy categories [76]. Yet this congruence question has not been systematically tested for Low-GI labeling in dairy choice settings. Consistent with H3, our results indicate that the Low-GI premium is larger in low-carbohydrate profiles, suggesting a cue congruence mechanism: “low carb” (a quantity cue) and “Low-GI” (a quality/rate cue) jointly form a more coherent health narrative and therefore increase the diagnosticity of the claim in that context.

We further find that the heterogeneity in carbohydrate-context effects is primarily driven by consumers who correctly understand the Low-GI concept, rather than by those who apply a more global, heuristic interpretation of the claim. This pattern supports a “decoder” mechanism: objective knowledge enables consumers to map the claim onto the product’s nutritional profile and adjust valuation in a context-sensitive way, rather than applying a blanket inference from the claim alone [10,14,33]. More broadly, our evidence reinforces prior findings that consumers’ understanding of health claims is a key determinant of how such information is used in evaluation and choice [77]. It also aligns with yogurt-specific evidence showing that consumers’ valuation of claims depends on how claims are interpreted jointly with product nutrition characteristics [74], and with process evidence indicating that attention and information use shape preferences for nutrition claims [66]. At the same time, recent yogurt evidence suggests that the effect of simplified FOP health labeling on WTP may vary and can even be negative in some settings, underscoring the importance of understanding how consumers interpret label meanings rather than assuming uniform effects [78]. Extending this line of work, we not only focus on the Low-GI claim as a physiologically complex cue, but also combine claim understanding with an explicit nutritional scenario (carbohydrate context) to identify how knowledge enables context-sensitive valuation—an interaction that, to our knowledge, has not been directly monetized in prior dairy labeling research.

For knowledgeable consumers, three patterns are particularly informative. First, WTP is strongest when “Low-GI” is paired with low carbohydrate content, consistent with a reinforcing two-cue interpretation (a favorable quantity–quality bundle). Second, knowledgeable consumers retain a positive valuation even in high-carbohydrate profiles, consistent with a risk-mitigation logic; Low-GI may be interpreted as informative about the rate of postprandial glycemic response relative to higher-GI alternatives, even when carbohydrate quantity is high [5,6]. Third, knowledgeable consumers discount the claim in the regular-carbohydrate reference context, where functional positioning is less diagnostic and the incremental informational value of the claim is weaker. By contrast, less knowledgeable consumers display a more global premium across contexts, consistent with a heuristic “health halo” response rather than mechanism-based evaluation [10,33]. Taken together, these results provide direct support for H4 by showing that objective Low-GI knowledge functions as a cognitive decoder that enables differentiated valuation of the label across nutritional contexts.

### 5.4. Implications for Industry and Policy

Our evidence suggests that the behavioral effectiveness of a Low-GI claim depends on whether consumers can interpret it correctly and integrate it with co-occurring nutrition information. This supports approaches that reduce comprehension costs—clearer claim definitions, more standardized presentation, and complementary interpretive elements—which help consumers to understand what the claim does and does not imply [2,10].

The results reveal segmentation opportunities that align tightly with consumer behavior. For low-carbohydrate products, Low-GI can be positioned as a complementary “quality-of-carbohydrate” signal that reinforces an already coherent nutrition profile. For higher-carbohydrate products, the claim may be framed more carefully as an absorption-rate cue that differentiates the product within its category, without implying that Low-GI negates high carbohydrate quantity [5,6]. More broadly, firms’ returns to Low-GI labeling are likely to be highest when targeting consumers with higher health orientation and when the product formulation supports a credible nutrition narrative [14,15].

From a policy perspective, our findings imply that the welfare impact of physiological claims is conditional on consumer capability. Motivation raises general responsiveness, but knowledge determines whether the claim is used diagnostically [33]. Therefore, policy packages that combine claim standardization and enforcement with targeted nutrition education are more likely to translate labeling into informed choices [2,14].

### 5.5. Limitations and Future Research

Several limitations point to promising directions for future research in food labeling and consumer behavior. First, as a stated preference DCE, our WTP estimates may differ from real market behavior under time pressure, budget constraints, and habitual purchasing; future work should validate these patterns using incentive-compatible designs, scanner/transaction data, and field evidence. Second, our sample was recruited through a non-probabilistic approach and reflects a limited geographical scope, which may constrain external validity and the generalizability of the estimated WTP premia and heterogeneity patterns; replication with probability-based samples and broader regional coverage would strengthen inference. Third, we focused on one product category (plain yogurt). Because label interpretation depends on the food matrix and category expectations, replication in other contexts would clarify how general the “cue reinforcement vs. risk mitigation” logic is [17]. Fourth, our objective knowledge measure captures concept recognition (i.e., identifying what Low-GI means) rather than the depth or breadth of Low-GI knowledge, nor does it capture broader label trust or subjective understanding; incorporating multi-item or graded knowledge scales, alongside measures of trust and perceived comprehension, would help distinguish “knowing” from “believing” a claim and could reveal more nuanced gradients in context-sensitive valuation [10,33]. Finally, more policy-evaluation work could exploit natural experiments or staggered introductions of labeling rules to assess causal impacts on purchasing and diet quality.

## 6. Conclusions

This study used a discrete choice experiment (DCE) to quantify consumers’ willingness to pay (WTP) for a “Low-GI” front-of-pack (FOP) claim in China’s yogurt market and to unpack why this value varies across consumers and product profiles. Positioned within the broader agenda of food labeling as a policy instrument, our findings highlight that complex physiological claims do not function as unconditional signals of healthiness. Instead, their behavioral and welfare relevance depends on (i) whether consumers are motivated to use health information (health orientation) and (ii) whether they have the label-specific capability to interpret what the claim means (objective Low-GI knowledge), especially when other nutrition cues are present on the package. In other words, the effectiveness of emerging labels hinges on a “motivation–capability” pathway, and the label’s economic value is structurally shaped by the interaction between the claim and the surrounding nutrition context.

## Figures and Tables

**Table 1:** Attributes and levels used in the discrete choice experiment (DCE).

Attribute	Levels	Unit/Description
Low-GI label	Absent; Present	Front-of-pack label shown as an icon. In the questionnaire, the items were presented as follows: 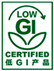 Prior to the DCE, respondents were shown the Low-GI logo and informed that it denotes a Low-GI label; no additional information about health benefits was provided.
Carbohydrate content	Low; Regular; High	Values per 100 g of yogurt (auxiliary numeric information).
Fat content	Skim; Low-fat; Whole-fat	Values per 100 g of yogurt (auxiliary numeric information).
Organic label	Absent; Present	Certification label shown as an icon. In the questionnaire, the items were presented as follows: 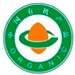 Before the DCE began, we showed respondents this image and informed them that it represents an organic label.
Price	2; 4.5; 8	CNY per 135 g serving.

Notes: Approximate nutrition values were provided as auxiliary information on the choice cards to facilitate interpretation of the attribute profiles. Specifically, carbohydrate content was shown as low (~6 g/100 g), regular (~9 g/100 g), and high (~12 g/100 g), and fat content was shown as skim (0 g/100 g), low-fat (~3 g/100 g), and whole-fat (~6 g/100 g). The attribute levels for price and nutritional content (carbohydrate and fat) were derived from a market survey of plain yogurt products on major Chinese e-commerce platforms to ensure market realism.

**Table 2:** Sample characteristics and opt-out behavior (*N* = 910).

Characteristic	Category	*n*	%
Gender	Male	456	50.110
	Female	454	49.890
Age	18–25	267	29.341
	26–35	197	21.648
	36–45	191	20.989
	46–55	185	20.330
	56–65	29	3.187
	>65	41	4.505
Education	Middle school or below	147	16.154
	High school/Vocational school	276	30.330
	Associate degree	254	27.912
	Bachelor’s degree	180	19.780
	Master’s degree	24	2.637
	Doctoral degree	13	1.429
	Other/Prefer not to say	16	1.758
Residence	Tier-1 city	140	15.385
	New Tier-1 city	134	14.725
	Tier-2 city	155	17.033
	Tier-3 or below/County-level	164	18.022
	Town/Rural area	162	17.802
	Other regions	155	17.033
Monthly Income (CNY)	<5000	488	53.626
	5000–9999	235	25.824
	10,000–19,999	93	10.220
	20,000–29,999	34	3.736
	30,000–49,999	34	3.736
	≥50,000	13	1.429
	Prefer not to say	13	1.429
Purchase Frequency (past month)	Never	105	11.538
	Once	250	27.473
	2–3 times	471	51.758
	≥4 times	84	9.231
Objective Low-GI knowledge	Selected the scientifically correct definition: a more gradual blood glucose rise given the same carbohydrate intake (ObjKnow = 1)	423	46.484
	Interpreted Low-GI as having lower carbohydrate or sugar content	256	28.132
	Interpreted Low-GI as involving less carbohydrate or sugar absorption by the body	182	20.000
	Reported not knowing the definition	49	5.385
		Mean	SD
Opt-out behavior	Opt-out rate	0.074	0.134
Health orientation	Index score	4.201	1.510
ObjKnow = 1	ObjKnow = 1 (correct definition)	0.465	0.499

Notes: Percentages are computed at the respondent level (*N* = 910). For the last two rows, columns report the mean and SD. Health orientation is the mean of three items on a 1–7 scale. Objective Low-GI knowledge was measured using a multiple-choice item; ObjKnow = 1 indicates selecting the scientifically correct definition. Opt-out rate is computed across respondents (12 choice tasks each).

**Table 3:** Mixed logit estimates for original-flavored yogurt choice.

Variable	Model 1	Model 2	Model 3
Panel A. Mean coefficients			
ASC_buy	1.859 *** (0.051)	1.907 *** (0.054)	1.900 *** (0.054)
Price	−0.094 *** (0.004)	−0.094 *** (0.005)	−0.094 *** (0.005)
Low-GI label	0.331 *** (0.028)	0.192 *** (0.050)	0.313 *** (0.053)
Organic label	0.152 *** (0.024)	0.150 *** (0.024)	0.150 *** (0.024)
Fat: skim	−0.193 *** (0.026)	−0.199 *** (0.026)	−0.202 *** (0.026)
Fat: whole	−0.324 *** (0.027)	−0.327 *** (0.027)	−0.330 *** (0.027)
Carb: low	0.175 *** (0.034)	0.035 (0.044)	0.030 (0.043)
Carb: high	−0.034 (0.033)	−0.074 * (0.045)	−0.081 * (0.044)
Low-GI × Carb: low	—	0.271 *** (0.058)	−0.014 (0.070)
Low-GI × Carb: high	—	0.068 (0.058)	−0.064 (0.070)
Low-GI × Health orientation	—	0.075 *** (0.023)	0.075 *** (0.023)
Low-GI × Objective knowledge	—	0.063 (0.059)	−0.222 *** (0.075)
ObjKnow × Low-GI × Carb: low	—	—	0.617 *** (0.086)
ObjKnow × Low-GI × Carb: high	—	—	0.307 *** (0.085)
Panel B. Standard deviations of random parameters			
SD: Low-GI label	0.181 *** (0.032)	0.056 (0.045)	0.066 (0.044)
SD: Fat skim	0.194 *** (0.034)	0.205 *** (0.034)	0.199 *** (0.035)
SD: Fat whole	0.208 *** (0.035)	0.195 *** (0.036)	0.185 *** (0.037)
SD: Organic	0.265 *** (0.028)	0.257 *** (0.029)	0.256 *** (0.029)
SD: Carb low	0.561 *** (0.038)	0.550 *** (0.038)	0.473 *** (0.040)
SD: Carb high	0.507 *** (0.038)	0.495 *** (0.038)	0.446 *** (0.039)
Model fit statistics			
Respondents	910	910	910
Choice tasks	10,920	10,920	10,920
Observations	43,680	43,680	43,680
Log likelihood	−13,539.629	−13,519.872	−13,494.501
AIC	27,137.26	27,105.74	27,059.00
BIC	27,389.11	27,392.34	27,362.96

Notes: Standard errors are in parentheses. * *p* < 0.10, *** *p* < 0.01.

**Table 4:** Willingness to pay for the Low-GI label across carbohydrate contexts.

Carbohydrate Context	ObjKnow = 0 WTP (SE) [95% CI]	ObjKnow = 1 WTP (SE) [95% CI]
Health orientation = −1 SD		
Regular	2.140 *** (0.619) [0.927, 3.353]	−0.233 (0.810) [−1.821, 1.356]
Low carb	1.991 *** (0.637) [0.742, 3.240]	6.212 *** (0.825) [4.596, 7.829]
High carb	1.459 ** (0.630) [0.225, 2.694]	2.373 *** (0.788) [0.827, 3.918]
Health orientation = Mean (0, centered)		
Regular	3.346 *** (0.583) [2.204, 4.487]	0.973 (0.630) [−0.262, 2.209]
Low carb	3.197 *** (0.610) [2.001, 4.393]	7.418 *** (0.682) [6.082, 8.754]
High carb	2.665 *** (0.589) [1.511, 3.819]	3.579 *** (0.616) [2.372, 4.785]
Health orientation = +1 SD		
Regular	4.552 *** (0.763) [3.056, 6.048]	2.179 *** (0.652) [0.902, 3.457]
Low carb	4.403 *** (0.791) [2.852, 5.954]	8.624 *** (0.732) [7.189, 10.059]
High carb	3.871 *** (0.764) [2.375, 5.368]	4.785 *** (0.651) [3.509, 6.060]

Notes: WTP is in CNY and computed as −ΔU/β_price (delta method). Standard errors are in parentheses below point estimates; brackets report 95% confidence intervals. ** *p* < 0.05, *** *p* < 0.01.

**Figure A1:**
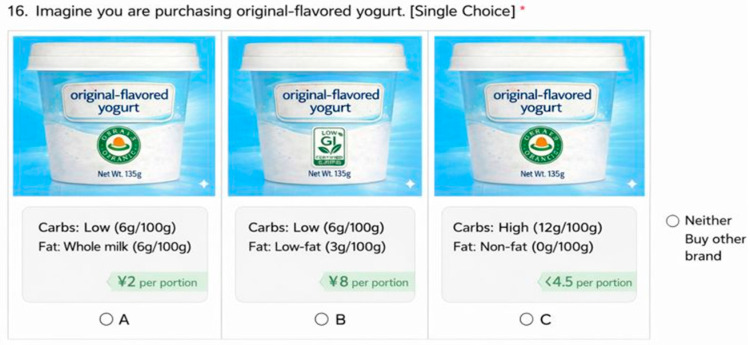
Illustrative English translation of the decision situation shown in Figure 1. The actual experiment was conducted in Chinese; this translation is provided for reference. * indicates a mandatory question.

## Data Availability

The data and analysis code presented in this study are available from the corresponding author on reasonable request.

## Abbreviations

The following abbreviations are used in this manuscript:

FOP	Front-of-pack
GI	Glycemic Index
WTP	Willingness to Pay
DCE	Discrete Choice Experiment
RUT	Random Utility Theory
MIXL	Mixed Logit
ASC	Alternative-Specific Constant
CNY	Chinese Yuan
AIC	Akaike Information Criterion
BIC	Bayesian Information Criterion
SD	Standard Deviation
SE	Standard Error
